# Branched-Chain Fatty Acids—An Underexplored Class of Dairy-Derived Fatty Acids

**DOI:** 10.3390/nu12092875

**Published:** 2020-09-20

**Authors:** Victoria M. Taormina, Allison L. Unger, Morgan R. Schiksnis, Moises Torres-Gonzalez, Jana Kraft

**Affiliations:** 1 Department of Nutrition and Food Sciences, The University of Vermont, Burlington, VT 05405, USA; Victoria.Taormina@uvm.edu; 2 Department of Animal and Veterinary Sciences, The University of Vermont, Burlington, VT 05405, USA; Allison.Unger@uvm.edu (A.L.U.); Morgan.Schiksnis@uvm.edu (M.R.S.); Jana.Kraft@uvm.edu (J.K.); 3 National Dairy Council, Rosemont, IL 60018, USA; 4 Department of Medicine, Division of Endocrinology, Metabolism and Diabetes, The University of Vermont, Colchester, VT 05446, USA

**Keywords:** *anteiso*, branched-chain amino acids, cancer, diabetes, inflammation, *iso*, metabolic diseases, milk, phytanic acid

## Abstract

Dairy fat and its fatty acids (FAs) have been shown to possess pro-health properties that can support health maintenance and disease prevention. In particular, branched-chain FAs (BCFAs), comprising approximately 2% of dairy fat, have recently been proposed as bioactive molecules contributing to the positive health effects associated with the consumption of full-fat dairy products. This narrative review evaluates human trials assessing the relationship between BCFAs and metabolic risk factors, while potential underlying biological mechanisms of BCFAs are explored through discussion of studies in animals and cell lines. In addition, this review details the biosynthetic pathway of BCFAs as well as the content and composition of BCFAs in common retail dairy products. Research performed with in vitro models demonstrates the potent, structure-specific properties of BCFAs to protect against inflammation, cancers, and metabolic disorders. Yet, human trials assessing the effect of BCFAs on disease risk are surprisingly scarce, and to our knowledge, no research has investigated the specific role of dietary BCFAs. Thus, our review highlights the critical need for scientific inquiry regarding dairy-derived BCFAs, and the influence of this overlooked FA class on human health.

## 1. Introduction

Milk and dairy products have been a staple of the human diet for thousands of years [1] and represent one of the most important agricultural commodities in regard to human nutrition. Dairy products have been instituted in most dietary guidelines around the world as they provide a large variety of essential nutrients and several key shortfall nutrients. Moreover, milk and dairy products hold a unique position among all foods as they are considered the largest single source of natural bioactive components [2]. In particular, dairy fat is the most complex and diverse dietary fat source in nature comprised of an impressive fatty acid (FA) repertoire (>400 different FAs and FA derivatives [3]) that accounts for the myriad of nutritional, organoleptic, and technological characteristics of milk and dairy products. Specifically, dairy fat contains a unique variety of bioactive FAs that are synthesized by rumen microbes (i.e., bacteria and protozoa) and the mammary gland. These FAs consist of short- and medium-chain FAs (4 to 13 carbons), positional and geometric isomers of octadecanoate (18:1), conjugated linoleic acids, odd-chain FAs (15:0 and 17:0), and branched-chain FAs (BCFAs). Of the repertoire of FAs in dairy fat, about 14% of them are these unique dairy-derived FAs [4] and several are known to impact normal mammalian physiology by functioning as bioactive molecules, exerting beneficial properties that support health and well-being [5,6,7]. To date, most research efforts in the arena of dairy FAs have been centered on the biological effects of conjugated linoleic acids, specifically rumenic acid (18:2 *c*9,*t*11), which was originally driven by the discovery of its demonstrated anticarcinogenic activity [8]. However, far less work has focused on other bioactive dairy-derived FAs.

BCFAs are an emerging group of bioactive FAs sparking growing research interest within the scientific community due to their biological effects and potential pro-health benefits. Because BCFAs are principally derived from rumen bacteria, milk and dairy products pose unique dietary sources of BCFAs. The objectives of this paper are to review and summarize the current knowledge on BCFAs and specifically to evaluate the possible role of dairy-derived BCFAs in human health.

## 2. Structure and Origin of BCFAs in Ruminants

**Figure 1:**
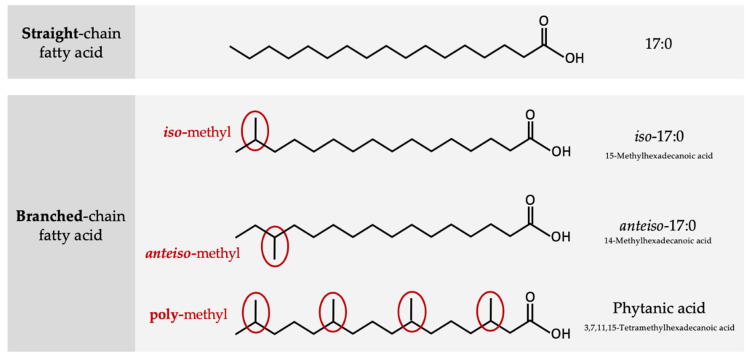
Structural differences between straight chain fatty acids and *iso-*, *anteiso-*, and multimethyl branched-chain fatty acids.

BCFAs are commonly saturated FAs substituted with one (mono-) or more (di-/poly-) methyl branch(es) on the carbon chain. Typically, BCFAs possess either an *iso* structure where the FA has the branch point on the penultimate carbon atom (i.e., one from the end) or an *anteiso* structure where the branch point is located on the antepenultimate carbon atom (i.e., two from the end; Figure 1). More than 50 BCFAs have been identified in ruminant-derived fats [9,10] but *iso*- and *anteiso*-mono-methyl BCFAs with chain lengths from 14 to 17 carbon atoms are quantitatively the most abundant BCFAs in milk fat [4,11,12,13,14,15,16].

**Figure 2:**
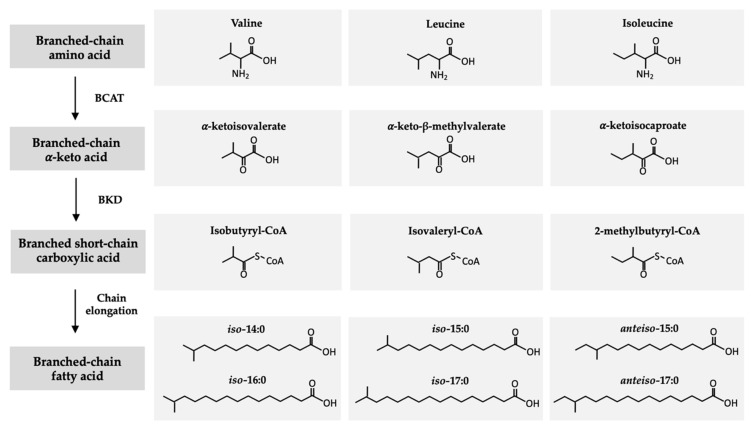
Biosynthetic pathway of branched-chain fatty acids from branched-chain amino acids. BCAT: branched-chain amino acid transferase (BCAT) enzyme. BKD: branched-chain-α-ketoacid dehydrogenase.

In ruminants, monomethyl BCFAs are synthesized by the microorganisms that reside within the rumen, specifically bacteria and protozoa [17], which utilize dietary branched-chain amino acids (BCAAs), i.e., valine, leucine, and isoleucine, to form BCFAs (Figure 2) [18]. Through a common biosynthetic pathway, BCAAs are first transformed into branched-chain α-ketoacids through the removal of the amino group by a BCAA transferase enzyme [19]. These α-ketoacid products, α-ketoisovalerate, α-keto-β-methylvalerate, and α-ketoisocaproate, are subsequently decarboxylated by branched-chain-α-ketoacid dehydrogenase producing the respective branched short-chain carboxylic acids isobutyral-CoA, isovaleryl-CoA, and 2-methylbutyral-CoA [20]. Finally, branched short-chain carboxylic acids are elongated by BCFA synthetase, with malonyl-CoA as the chain extender, to form *iso-* and *anteiso-*BCFAs [18]. The products of the BCFA biosynthetic pathway are *iso*-14:0 and *iso*-16:0, derived from valine, *iso*-15:0 and *iso*-17:0 from leucine, and *anteiso*-15:0 and *anteiso*-17:0 from isoleucine. 

**Figure 3:**
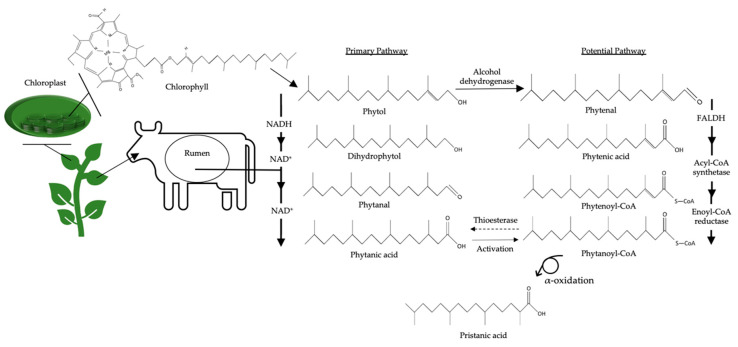
Synthesis of phytanic and pristanic acid via rumen microorganisms derived from chlorophyll within forages NAD: Nicotinamide adenine dinucleotide. NAD^+^: the oxidized form of NAD. NADH: the reduced form of NAD (protonated with a hydrogen).

Phytanic acid (3,7,11,15-tetramethylhexadecanoic acid) and its metabolite pristanic acid (2,6,10,14-tetramethylpentadecanoic acid), two multimethyl BCFAs, are also predominantly synthesized in the rumen (Figure 3) [21,22]. Phytanic acid is derived from the phytol component of chlorophyll found in forages [21]. While mammals are unable to cleave the ring structure from the phytol moiety of chlorophyll, bacteria release phytol in the rumen [23]. The primary synthesis pathway of phytanic acid in ruminants is initiated by the biohydrogenation of phytol to produce dihydrophytol [21,24]. Dihydrophytol is subsequently converted to phytanal before it is synthesized into phytanic acid [21,24]. Notably, a potential secondary synthesis pathway has been described in non-ruminant tissues and marine bacteria [22,24,25] and may be applicable to ruminant animals as well, although there is only limited evidence [26]. In this case, phytol is first converted to phytenal by an alcohol dehydrogenase [22]. Fatty aldehyde dehydrogenase is used to transform phytenal to phytenic acid before it is modified into phytenoyl-CoA by an acyl-CoA synthetase. Subsequently, phytenoyl-CoA is converted by an enoyl-CoA reductase to form phytanoyl-CoA [24,25]. The final synthesis step of phytanoyl-CoA to phytanic acid is thought to occur through α-oxidation [25] due to the enzymatic activity of thioesterase, a protein involved in α-oxidation [24]. In order to form pristanic acid, phytanic acid is activated by phytanoyl-CoA synthetase to create phytanoyl-CoA, which can then undergo α-oxidation to produce pristanic acid.

BCFAs are key structural lipid constituents of the bacterial membranes [17] where they play an important regulatory role in membrane fluidity and permeability [27]. Importantly, bacterial membrane lipids make an important contribution to ruminant milk fat, as bacterial cells leaving the rumen pass to the duodenum where their membrane FAs are absorbed and subsequently incorporated into milk fat and other tissues [21].

## 3. Occurrence and Metabolism of BCFAs in Humans

BCFAs are common constituents of microbial lipids present in abundant quantities but have also been found in many other organisms (e.g., *C. elegans*, [28]) including mammals [29,30,31], although in much lower amounts. In humans, BCFAs have been detected in various tissues and fluids including vernix caseosa, the biofilm covering the skin of the fetus [32,33], colostrum and mature breast milk [34,35,36], adipose tissue [37], and serum [38]. Early work by Nicolaides and co-authors [39,40,41] established that the meibomian and sebaceous glands of the human skin produce BCFAs secreted into meibum and sebum, respectively. Subsequent research in vitro and in vivo using mouse models confirmed the endogenous synthesis of BCFAs from their respective BCAA precursors (i.e., valine, leucine, isoleucine) as a result of the BCAA catabolic pathway [30,31,42,43]. BCAA degradation in humans is comparable to that in bacteria (Figure 2). Despite the endogenous synthesis, dietary intake of BCFAs is presumably the principal source of BCFAs in the human body. 

The intake of BCFAs depends on the type of food and the fat content of the food consumed. While ruminant-derived foods (i.e., milk and meat and their respective products) are the chief source of BCFAs, fish and non-dairy fermented foods (e.g., kimchi, sauerkraut, miso, tempeh) may also supply BCFAs, although only at very small quantities. The mean daily intake of BCFAs in the United States has been estimated to range between 220 mg/day (for milk only [44]) and 500 mg/day (from dairy and beef products combined [11]). The contribution of total dairy products to the BCFA intake has been calculated at 317 mg/day [11]. There is no requirement for BCFAs per se but it is conceivable that the daily dietary intake of BCFAs needs to be higher than the current estimates to achieve potential health benefits.

Very little is known about the fate and metabolism of dietary BCFAs in humans, thus representing an important area for future research. While information is scarce, it can be presumed that digestion, absorption, and transport of BCFAs in humans is comparable to that of other long-chain FAs. Our knowledge of BCFA uptake by cells is based largely on work in cell lines, specifically from the intestine (Caco-2), fetal small intestine (H4), and breast cancer (SKBR-3 and MCF-7), following exposure to one or a mixture of BCFAs [45,46,47,48,49]. From this limited perspective, it is thought that the cellular uptake of BCFAs is dependent on BCFA length and configuration. Using Caco-2 cells, Yan et al. [45] demonstrated that BCFAs can be taken up and further metabolized into their elongation or chain-shortened products. Other work indicates that in fetal intestinal cells incubated with identical concentrations of BCFAs *(*i.e., *iso*-16:0, *anteiso*-17:0, *iso*-18:0, and *iso*-20:0), *anteiso*-17:0 exhibited the greatest uptake efficiency, followed by *iso*-16:0, *iso*-18:0, then *iso*-20:0, with significant differences, and a specific hierarchy, between these BCFAs [47]. In contrast, MCF-7 human breast cancer cells accumulated greater amounts of *iso*-15:0 and *iso*-17:0 compared to their *anteiso* analogs [49]. Dietary supplementation of BCFAs to neonatal rat pups demonstrated that BCFAs incorporate into ileal phospholipids as well as liver tissue and serum [50]. Research conflicts as to whether BCFAs are preferentially incorporated into cellular triacylglycerols or phospholipids [47,48], and whether the FA structure plays a determinant role requires further investigation. 

## 4. Occurrence of BCFAs in Dairy Products

### 4.1. Content and Composition of BCFAs in Milk

Within dairy foods, research to date has mainly focused on the content and composition of BCFAs derived from cow’s milk. From this work, studies established that cow’s milk is a significant source of BCFAs, typically comprising 1.7–3.4% BCFAs of total FAs (Table 1). When considered on a per serving basis, three servings of whole milk (3.25% milk fat) per day can thus provide 367–763 mg of BCFAs (Table 1). In milk fat, monomethyl BCFAs are the principal BCFAs present, with a chain length of 14–17 carbons and the methyl group occurring in either an *iso* or *anteiso* configuration. In general, total *iso*- and *anteiso*-BCFAs isomers occur in an approximate ratio of 1:1 (Table 1). Short-chain (4:0–6:0) and medium-chain (7:0–13:0) *iso*- and *anteiso*-BCFA isomers are also present in cow’s milk but in rather a minor abundance, accounting for approximately 0.01 and 0.12% of total BCFAs, respectively [51].

Multimethyl BCFAs, namely phytanic and pristanic acid, are consistent constituents within milk fat, but are often overlooked or ignored, likely because of their very low content and analytical limitations. In cow’s milk, the content of phytanic acid typically ranges between 0.10 and 0.50% of total FAs, equivalent to 7–37 mg/serving whole milk [12,23,52,53,54,55,56]. The presence of phytanic acid in milk is specifically dependent on the feed type of the dairy cow, with higher amounts associated with the intake of grass-based rations rich in chlorophyll [23,52,53,55]. Pristanic acid, a metabolite of phytanic acid, occurs in very low amounts in milk fat, at approximately 0.04–0.06% (3–4 mg/serving whole milk) [23]. 

### 4.2. BCFAs in Milk across Ruminant Species

A survey of the recent literature regarding the BCFA composition of ruminant milk shows that milk from sheep and goats comprises approximately 1.8–3.1% and 1.2–2.4% BCFAs of total FAs, respectively, and is thus comparable to cow’s milk (1.7–3.4% BCFAs of total FAs; Table 1). Yet, the content of total and individual BCFAs in milk varies considerably based on the ruminant species of origin (Table 2). In particular, on a per serving basis, milk from sheep contains considerably more BCFAs per serving compared to milk from cows (204–502 mg/serving versus 123–254 mg/serving). In the U.S., cow’s milk is standardized to 3.25% milk fat (i.e., whole milk), however, this practice is less common for specialty milk such as goat and sheep milk. Differences in the BCFA content of ruminant-derived milks therefore appear to be chiefly reflective of milk fat standardization practices in retail milk, rather than species differences in BCFA occurrence. 

### 4.3. Comparison of BCFAs among Dairy Products

The content of BCFAs in dairy also differs depending upon the type of dairy product (Table 2). For example, Ran Ressler et al. [11] found that yogurt contained an equivalent of 152 mg BCFAs/serving, while butter contained an equivalent of 195 mg/serving. Limited work assessing the BCFA content in cheese demonstrates that cheese type appears to be an important consideration as well (82–322 mg/serving depending upon cheese variety). One notable source of variation in the content of BCFAs per serving among cheese types is the differing fat content. For example, one serving (28.35 g) of low-moisture Mozzarella (22.1% fat) contains 82 mg BCFAs, whereas Cheddar (33.0% fat) contains 148 mg BCFAs [11]. One serving of butter (14.2 g), another dairy-derived product high in fat (<80%), can contain more than 200 mg of BCFAs (137–204 mg/serving; Table 2).

## 5. Human Trials Assessing Health Effects of BCFAs

To date, epidemiological research indicates that dairy fat consumption is neutral or protective against cardiometabolic diseases. However, the specific role of BCFAs is less understood, as these studies largely rely upon either food frequency questionnaires and/or the measurement of specific dairy FA biomarkers (i.e., 15:0, 17:0, and 16:1 *t*9) in blood or tissues [57,58,59,60], but not BCFAs.

### 5.1. Obesity

Epidemiological studies have noted that the intake of full-fat dairy products can be beneficial for long-term weight maintenance [61,62]. Other research which specifically focused on the consumption of BCFAs suggests that BCFAs have an important role in energy homeostasis. For example, one study found that total BCFAs in serum were higher in non-obese women than obese women and that *iso*-BCFAs were inversely associated with body mass index [38]. Similar results have been reported in a recent study by Pakiet et al. [63]. Another study found that the adipose tissue of lean subjects had higher proportions of total BCFAs and individual BCFAs than obese individuals [37]. 

One niche of scientific interest is the relationship between tissue concentrations of BCFAs and weight status in individuals post-gastric bypass surgery. In a longitudinal study, the BCFAs of adipose tissue in obese subjects was assessed at baseline and one year following Roux-en-Y gastric bypass surgery [37]. This study demonstrated that, after one year, the proportion of total BCFAs increased after surgery-induced weight loss. A follow-up study found similar results, showing that serum BCFAs increased in individuals after one-anastomosis gastric bypass surgery [63]. Of note, a similar study found no changes in serum BCFAs two weeks after one-anastomosis gastric bypass surgery, suggesting that a minimum time period may be needed to observe changes [64]. 

### 5.2. Insulin Sensitivity

Serum collected from fasted individuals showed a modest inverse correlation between serum BCFA concentrations and an index of insulin resistance (homeostatic model of assessment of insulin resistance), suggesting that BCFAs may promote insulin sensitivity [63]. Similarly, total BCFAs in adipose tissue have been associated with measurements of insulin sensitivity (i.e., insulin-stimulated glucose rate of disappearance) [37]. Moreover, Mika et al. [38] observed that two BCFAs, *anteiso-*15:0 and *iso-*17:0, were negatively correlated with fasted serum insulin levels but not with the homeostatic model assessment of insulin resistance.

### 5.3. Limitations of Current Evidence Available in Humans

Research suggests that BCFAs promote weight maintenance and metabolic health [37,38,63], however, more studies are needed to ascertain the specific impact of dietary BCFAs, and to what extent dietary BCFAs contribute to these outcome measures. Research assessing the metabolic effects of dairy-derived BCFAs is still lacking. Therefore, for this review, we considered studies evaluating the role of BCFAs on health, regardless of the source. While dietary patterns are the plausible source of variation in tissue BCFA occurrence in humans, BCFAs derived from an individual’s gut microbiota or metabolism from BCAAs cannot be discounted. Future work evaluating the relationship between diet and disease risk should consider the inclusion of BCFAs as biomarkers of dairy fat consumption.

## 6. Role of Dairy-Derived BCFAs in Health: Potential Mechanisms

### 6.1. Inflammation

Inflammation is an important underlying factor contributing to the pathogenesis of many metabolic diseases [94,95,96]. In particular, gastrointestinal health is critical for proper immune function and regulation of inflammation [97,98]. Research utilizing gastrointestinal cell lines has demonstrated the potent anti-inflammatory potential of dietary BCFAs. For example, exposure of Caco-2 cells to BCFAs reduced the lipopolysaccharide-induced gene expression of important proinflammatory mediators (i.e., IL-8, TLR-4, and NF-κB) [45]. In addition, certain shorter chain BCFAs (*i.e*., *iso*-14:0, *iso*-16:0, *anteiso*-13:0) improved the lipopolysaccharide-induced decrease in cell viability. Similar results were reported in a follow-up study [46] when Caco-2 cells were treated with monoacylglycerols or free FAs comprised of ~30% BCFAs isolated from the vernix. 

Animal work is limited, but also supports that BCFAs attenuate inflammation and related diseases. Ran-Ressler et al. [50] examined the effect of a diet containing a mixture of BCFAs (*iso*-14:0, *anteiso-*15:0, *iso-*16:0, *anteiso*-17:0, *iso*-18:0, and *iso*-20:0) on necrotizing enterocolitis in neonatal Sprague–Dawley pups. BCFA-fed pups had a lower incidence of necrotizing enterocolitis and an enhanced gene expression of IL-10, an anti-inflammatory cytokine. Moreover, the cecal samples of pups fed the BCFA-enriched rat formula had a greater abundance of *Bacillus subtilis* and *Pseudomonas aeruginosa* compared to those fed standard rat formula, which is notable as both are known to contain BCFAs in their membranes [18,50,99]. Of note, an inverse association has been found in humans for serum *iso*-BCFAs (i.e., *iso-*15:0, *iso-*16:0, *iso-*17:0, and *anteiso-*15:0) and serum C-reactive protein [38], an important inflammatory marker for type 2 diabetes risk.

### 6.2. Anticarcinogenic Properties

One of the first beneficial effects attributed to BCFAs were their anticancer properties discovered 20 years ago. Specifically, *iso-*15:0 is known to inhibit the growth of T-cell non-Hodgkin lymphoma (Jurkat, Hut78, and EL4 cell lines [100]) and various types of carcinoma cell lines including, breast (MCF-7 and SKBR-3), prostate (DU145), lung (NCI-H1688), pancreas (BxPC-3), liver (SNU-423), bladder (T24, 5637, and UM-UC-3), leukemia (K-562), and gastric (NCI-SNU-1) and colorectal carcinoma (HCT 116) in a dose- and/or time-dependent manner by inhibiting proliferation and inducing apoptosis [48,49,101,102]. In a comparison of eight *iso*-BCFA species of varying carbon chain lengths (*iso*-12:0 through *iso*-20:0), Wongtangtintharn et al. [103] demonstrated that the anticarcinogenic activity of BCFAs are dependent on the chain length, with *iso*-16:0 exerting the highest cytotoxic activity. Furthermore, a more recent study showed that BCFAs with an *iso* structure (i.e., *iso*-15:0 and *iso*-17:0) were more potent than their *anteiso* counterparts (i.e., *anteiso*-15:0 and *anteiso*-17:0) [49]. Similarly, in vivo experiments have shown that BCFAs inhibit tumor growth in both mouse xenograft tumor models in which cancer cells were co-grafted [100,101] and in the orthotopic VX2 squamous cell carcinoma model in rabbits [104].

BCFAs may also induce beneficial, location-specific effects on angiogenesis. Treatment with *iso-*15:0 promoted angiogenesis post cerebral ischemia/reperfusion injury [105], while *anteiso*-15:0 suppressed angiogenesis to aid corneal recovery [106]. These effects appear to be structure specific, but studies in this area are still very limited, hence more research is needed. Moreover, the biological significance of the role of BCFAs on angiogenesis in the context of cancer growth is not yet established.

### 6.3. Energy and Glucose Homeostasis

Previous research has suggested that the body content, and specifically the liver content, of BCFAs reflects the ingestion of their respective BCAA precursors [30,107]. Furthermore, Brooks et al. [107] and Garcia-Caraballo et al. [30] observed that the body or hepatic content of BCFAs were inversely correlated with the body triacylglycerol content, as shown in *C. elegans*, or the liver triacylglycerol content, as shown in mice, respectively. Likewise, a recent in vitro study, using a human fatty liver cell line model (i.e., L02 cells), concluded that individual BCFAs (*iso-*15:0 and *iso-*18:0) reduced the cellular triacylglycerol content and were associated with an upregulation of multiple genes involved in lipid catabolism [108]. These observations are in accordance with human trials which showed that, in serum, total BCFAs [63] and specific *iso*-BCFAs [38] had an inverse relationship with triacylglycerols. Thus, the results from these studies indicate that BCFA may be directly or indirectly involved in the regulation of fat storage in the body. 

As described above, BCFAs may favorably influence insulin sensitivity in humans, however, studies assessing potential mechanisms involved are scarce. One study performed with rat insulinoma INS-1 β-cells demonstrated that *iso-*17:0 may beneficially modulate β-cell function via the upregulation of Pdx1 and PPAR-γ transcription factors [109]. Yet, more in vivo research is necessary to confirm these results. 

### 6.4. Biological Functions of Polymethyl BCFAs: Phytanic Acid

The health effects of dietary phytanic acid, both positive and negative, have been extensively examined in a recent review [110]. In particular, Roca-Saavedra et al. [110] summarize the biological complications that arise due to an excess of phytanic acid within the body, such as neurological injury, oxidative stress, and cancers. To that end, recent in vitro work continues to show that phytanic acid, when provided at concentrations exceeding normal physiological ranges, is neurotoxic [111,112]. Importantly, the nutritional relevance of such studies is called into question for the general population, as the accumulation of phytanic acid due to impaired lipid metabolism (e.g., Refsum disease) is thought to be rare in terms of prevalence [113]. Nakanishi et al. [114] demonstrated that when cells are treated with concentrations of phytanic acid comparable to that found in the plasma of healthy individuals, phytanic acid exerted beneficial immunomodulatory effects in a PPAR-α-dependent manner. Furthermore, there is no clinical evidence that the consumption of phytanic acid in naturally occurring amounts, e.g., phytanic acid derived from milk and dairy products, detrimentally impacts health in individuals with normal lipid metabolism. 

Roca-Saavedra et al. [110] also describe favorable effects of phytanic acid on glucose and lipid metabolism, energy expenditure, as well as immune and anticancer functions. Indeed, efforts to ameliorate or prevent obesity have been gaining momentum, and phytanic acid has been attracting scientific interest as an active compound for potential novel therapeutic drugs. Emerging research identified phytanic acid as a potent agonist of PPAR-α [115], a transcription factor expressed in metabolically active tissues controlling cellular FA oxidation and regulating energy homeostasis [116]. An et al. [115] showed that phytol treatment in a high-fat diet-induced mouse model of obesity resulted in greater contents of phytanic acid in liver and brown adipose tissue as well as the activation of PPAR-α and its target genes. Additionally, a recent study reported that phytanic acid promotes beige adipogenesis in murine 3T3-L1 (white) adipocytes (also called browning) in a PPAR-α ligand-dependent manner [117]. Taken together, these novel findings add support to phytanic acid possibly playing a role in the regulation of energy homeostasis.

## 7. Conclusions

BCFAs are a class of saturated FAs that comprise a significant portion of total FAs in milk and dairy products. While humans can derive a limited amount of BCFAs from endogenous synthesis from BCAAs, dietary BCFAs remain the most important source of BCFAs within the body. Recent human trials indicate that BCFAs in tissues have a beneficial influence on metabolism. Mechanistic research validates these studies by demonstrating that BCFAs possess a wide range of structure-specific functions that may favorably influence health at the cellular and systemic level. Despite the promise of BCFAs as pro-health dietary constituents, our narrative review reveals a critical scarcity in the scientific understanding of the relationship between diet-derived BCFAs and disease risk. Of note, research has been slow to utilize these FAs as markers of dairy fat intake. Here we propose that BCFAs may be a useful and complimentary biomarker of dairy fat intake for future studies. It is clear that more research is needed to clarify the diverse biological roles of BCFAs in vivo, particularly through clinical and epidemiolocal studies that evaluate the relationship between BCFA consumption, food source, BCFA tissue concentrations, and disease.

## Figures and Tables

**Table 1:** Branched-chain fatty acid composition and content of cow’s milk.

FA ^1^	Proportion (% of Total FAs) ^2^	Content (mg/Three Daily Servings) ^3^
*iso*-13:0	0.02–0.03 [4,13,14,15] ^4^	5–7 [4,13,14,15] ^4^
*anteiso*-13:0	0.07–0.09 [4,13] ^4^	15–19 [4,13] ^4^
*iso*-14:0	0.08–0.22 [4,11,12,13,14,15] ^4^	18–48 [4,11,12,13,14,15] ^4^
*iso*-15:0	0.13–0.44 [4,11,12,13,14,15] ^4^	29–97 [4,11,12,13,14,15] ^4^
*anteiso*-15:0	0.37–0.93 [4,11,12,13,14,15] ^4^	81–206 [4,11,12,13,14,15] ^4^
*iso*-16:0	0.17–0.45 [4,11,12,13,14,15] ^4^	38–100 [4,11,12,13,14,15] ^4^
*iso*-17:0	0.26–0.56 [4,11,12,13,14,15] ^4^	58–123 [4,11,12,13,14,15] ^4^
*anteiso*-17:0	0.11–0.76 [4,11,12,13,14,15] ^4^	24–169 [4,11,12,13,14,15] ^4^
*iso-*18:0	0.01–0.09 [4,11,12,13,14,15] ^4^	2–20 [4,11,12,13,14,15] ^4^
Σ *iso-*BCFAs	0.87–1.75 [4,11,12,13,14,15] ^4^	193–387 [4,11,12,13,14,15] ^4^
Σ *anteiso-*BCFAs	0.67–1.69 [4,11,12,13,14,15] ^4^	149–375 [4,11,12,13,14,15] ^4^
Σ BCFAs ^5^	1.66–3.44 [4,11,12,13,14,15] ^4^	367–763 [4,11,12,13,14,15] ^4^

^1^ FA = fatty acid. ^2^ Averages were estimated by calculating the median of reported FA values when appropriate. ^3^ Content of FAs per serving was calculated from FA values listed (% of total FAs) as described by Bainbridge et al. [13], assuming 3.25% fat per serving whole milk, then multiplied by three. ^4^ Raw data obtained from previously published work [4]. ^5^ BCFAs = branched-chain FAs; sum of *iso* and *anteiso* BCFA isomers (13:0–18:0).

**Table 2:** Branched-chain fatty acid content (mg/serving) of common dairy products ^1^.

FA ^2^	Milk ^3^	Cheese ^4^			Yogurt ^5^	Butter ^6^	Sheep Milk ^7^	Goat Milk ^8^
		Soft/Semi-Soft ^9^	Semi-Hard/Hard ^10^	Unknown ^11^				
*iso-*13:0	2 [4,13,14,15] ^12^					2 [65]	8 [66]	1 [67]
*anteiso-*13:0	5–6 [4,13] ^12^					9 [65]	5 [66]	1–7 [67,68]
*iso-*14:0	6–16 [4,11,12,13,14,15] ^12^	5–14 [11,69]	6–20 [11,12,69]	56–13 [11,69]	9 [11]	8–18 [11,65,70]	8–24 [66,71,72]	5–10 [67,68,73,74]
*iso-*15:0	10–32 [4,11,12,13,14,15] ^12^	6–33 [11,69]	8–41 [11,12,69]	12–33 [11,69]	11 [11]	1–22 [11,65,70]	21–71 [66,71,72,75]	13–36 [67,68,73,74,76]
*anteiso-*15:0	27–69 [4,11,12,13,14,15] ^12^	26–68 [11,69]	33–87 [11,12,69]	35–62 [11,69]	47 [11]	36–67 [11,65,70]	45–122 [66,71,72,75]	25–47 [67,68,73,74,76]
*iso-*16:0	13–33 [4,11,12,13,14,15] ^12^	12–30 [11,69]	9–42 [11,12,69]	11–26 [11,69]	22 [11]	20–36 [11,65,70]	25–69 [66,71,72,75]	13–22 [67,68,73,74]
*iso-*17:0	19–41 [4,11,12,13,14,15] ^12^	8–41 [11,69]	8–52 [11,12,69]	14–38 [11,69]	19 [11]	22–34 [11,65,70]	34–115 [66,71,72,75]	20–58 [67,68,73,74,76]
*anteiso-*17:0	8–56 [4,11,12,13,14,15] ^12^	23–71 [11,69]	17–71 [11,12,69]	25–61 [11,69]	45 [11]	34–42 [11,65,70]	48–124 [66,71,72,75]	25–57 [67,68,73,74,76]
*iso-*18:0	1–7 [4,11,12,13,14,15] ^12^	<1–8 [11,69]	<1–8 [11,12,69]	<1–7 [11,69]	3 [11]	<1–7 [11,65,70]	17–20 [66,72]	5 [67]
Σ *iso-*BCFAs	64–129 [4,11,12,13,14,15] ^12^	32–127 [11,69]	32–164 [11,12,69]	53–116 [11,69]	64 [11]	68–106 [11,65,70]	108–255 [66,71,72,75]	53–96 [67,68,73,74,76]
Σ *anteiso-*BCFAs	50–125 [4,11,12,13,14,15] ^12^	51–139 [11,69]	50–158 [11,12,69]	68–123 [11,69]	91 [11]	69–107 [11,65,70]	96–247 [66,71,72,75]	50–104 [67,68,73,74,76]
Σ BCFAs ^13^	123–254 [4,11,12,13,14,15] ^12^	83–264 [11,69]	82–322 [11,12,69]	123–239 [11,69]	152 [11]	137–204 [11,65,70]	204–502 [66,71,72,75]	109–180 [67,68,73,74,76]

^1^ Averages were estimated by calculating the median of reported FA values when appropriate; content of FAs per serving was calculated from reported FA values (% of total FAs), assuming 93.3% of FAs in milk fat (correction for glycerol) [77]. ^2^ FA = fatty acid. ^3^ Cow-derived; based on the assumption of 3.25% fat, 244 g per serving [13]. ^4^ Cow-derived; based on reported % fat and serving sizes (g) listed by FoodData Central for each cheese variety [78,79,80,81,82,83,84,85,86,87,88,89] with the exception of Ricotta cheese (62 g/serving). ^5^ Cow-derived; based on reported % fat and serving size (245 g) listed by FoodData Central [90]. ^6^ Cow-derived; based on reported % fat (80%) and serving size (14.2 g) listed by FoodData Central [91]; when FA values were reported as μg/g butter [70], FAs per serving were calculated by assuming 14.2 g per serving [91] without correction factor for glycerol. ^7^ Based on reported % fat (median of values calculated when appropriate) or % fat listed by FoodData Central [92] if not reported; based on serving size (245 g) listed by FoodData Central [92]. ^8^ Based on reported % fat (median of values calculated when appropriate) or % fat listed by FoodData Central [93] if not reported; based on serving size (244 g) listed by FoodData Central [93]. ^9^ Includes Ricotta, Romadur, Cottage cheese, Camembert, Brie, Limburger, Feta, and Bavaria Blue. ^10^ Includes Provolone, low-moisture Mozzarella, Emmental, Cheddar, Montasio, Gouda, and Butter cheese (Butterkäse). ^11^ Includes Swiss, American, Alpine cheese, curd cheese, and Mozzarella (varieties without sufficient information reported to categorize). ^12^ Raw data underlying previously published work [4]. ^13^ BCFAs = branched-chain FAs; sum of *iso* and *anteiso* BCFA isomers (13:0–18:0).
